# The complete chloroplast genome sequence of the medicinal plant *Stephania epigaea* H. S. Lo, 1978 (Menispermaceae) from Yunnan, China

**DOI:** 10.1080/23802359.2022.2104670

**Published:** 2022-07-29

**Authors:** Qin Guan, Danping Feng, Min Fan

**Affiliations:** a College of Pharmacy, Dali University, Dali, China; b Key Laboratory of Yunnan Provincial Higher Education, Institute for Development of Yunnan Daodi Medicinal Materials Resources, Dali, China

**Keywords:** *Stephania epigaea*, complete chloroplast genome, phylogenetic analysis

## Abstract

*Stephania epigaea* H. S. Lo, 1978 is a medicinal plant commonly used in southwest China. This study characterized the first complete chloroplast (cp) genome sequence of this species. The complete cp was 157,738 bp in length, containing a large single-copy region (LSC) of 88,460 bp, a small single-copy region (SSC) of 19,778 bp, and a pair of inverted repeat regions (IRs) of 24,750 bp. It encoded 130 genes, including 85 protein-coding genes, 37 tRNA genes, and 8 rRNA genes. The GC content of the complete genome was 36.7%. Phylogenetic analysis of complete cp sequences revealed that *S. epigaea* was clustered with *S. japonica* from the Menispermaceae family.

*Stephania epigaea* H. S. Lo, 1978 (Menispermaceae) is distributed mainly in Yunnan and Sichuan provinces, China (Zhao et al. 2020; Dong et al. 2015), frequently used to treat cough, diarrhea, bellyache, injuries, and malaria (Xiao et al. 2021). Previous studies of this species mainly focused on its chemical constituents and medicinal properties (Lv et al. 2013; Dong et al. 2018). Two barcode analyses have been published using rDNA and a chloroplast marker (Xie et al. 2015; Wang et al. 2020). However, the complete chloroplast (cp) genome of this species has not yet been deciphered. Therefore, we reported the first complete cp genome of *S. epigaea*, which will provide a valuable resource for further genetic conservation, evolution and molecular breeding studies in the genus *Stephania.*

This article is licensed under the Regulations of Yunnan Province on Biodiversity Protection and is not require any ethical or institutional approvals. Healthy and fresh leaves of *S. epigaea* were collected from Dali county (25°84′ 95″ N, 100°11′6″ E) and deposited in the Herbarium of Dali University (http://yxy.dali.edu.cn/yhxy/, Min Fan, fanmin@dali.edu.cn) under the voucher number LJ2020060607. The total genomic DNA was extracted using the improved CTAB method (Doyle and Doyle 1986) and sequenced by the Illumina NovaSeq 6000 platform (Chang et al. 2021). In total, 19,098,862 clean reads (https://github.com/ndierckx/novoplasty) were de novo assembled by NOVOPlasty (Wang et al. 2020; Xia et al. 2021) and annotated by GeSeq with default settings (Tillich et al. 2017; Wei and Li 2021). The GenBank accession number is MZ678241.

The length of the *S. epigaea* cp genome was 157,738 bp with a typical quadripartite circular structure (Zhou et al. 2017), which contained a pair of inverted repeat regions (IRa and IRb, 24,750 bp), a large single-copy region (LSC, 88,460 bp), and a small single-copy region (SSC, 19,778 bp). All 130 genes were identified, including 85 protein-coding genes, 37 tRNA genes, and 8 rRNA genes. The overall GC content of the cp genome was 36.7%.

To evaluate the phylogenetic relationship of *S. epigaea*, 5 Menispermaceae, 10 Berberidaceae, and 9 Ranunculaceae complete cp genomes were downloaded from the NCBI database and aligned by MAFFT v7 (https://mafft.cbrc.jp/alignment/server/index.html) (Katoh and Standley 2013; Yamada et al. 2016). A maximum-likelihood tree with 1,000 bootstrap replicates was inferred using IQ-TREE v2.1.2 (Nguyen et al. 2015; Liu et al. 2021), with *Aristolochia debilis* (NC 036153.1) and *Aristolochia kwangsiensis* (NC 052833.1) as outgroups. The phylogenetic analysis revealed that *S. epigaea* was closely related to *S. japonica* (Figure 1). The cp genome of *S. epigaea* will be helpful for a comprehensive understanding of phylogenetic relationships among the genus *Stephania* and provide a valuable reference for the conservation genetics of this species.

**Figure 1.**
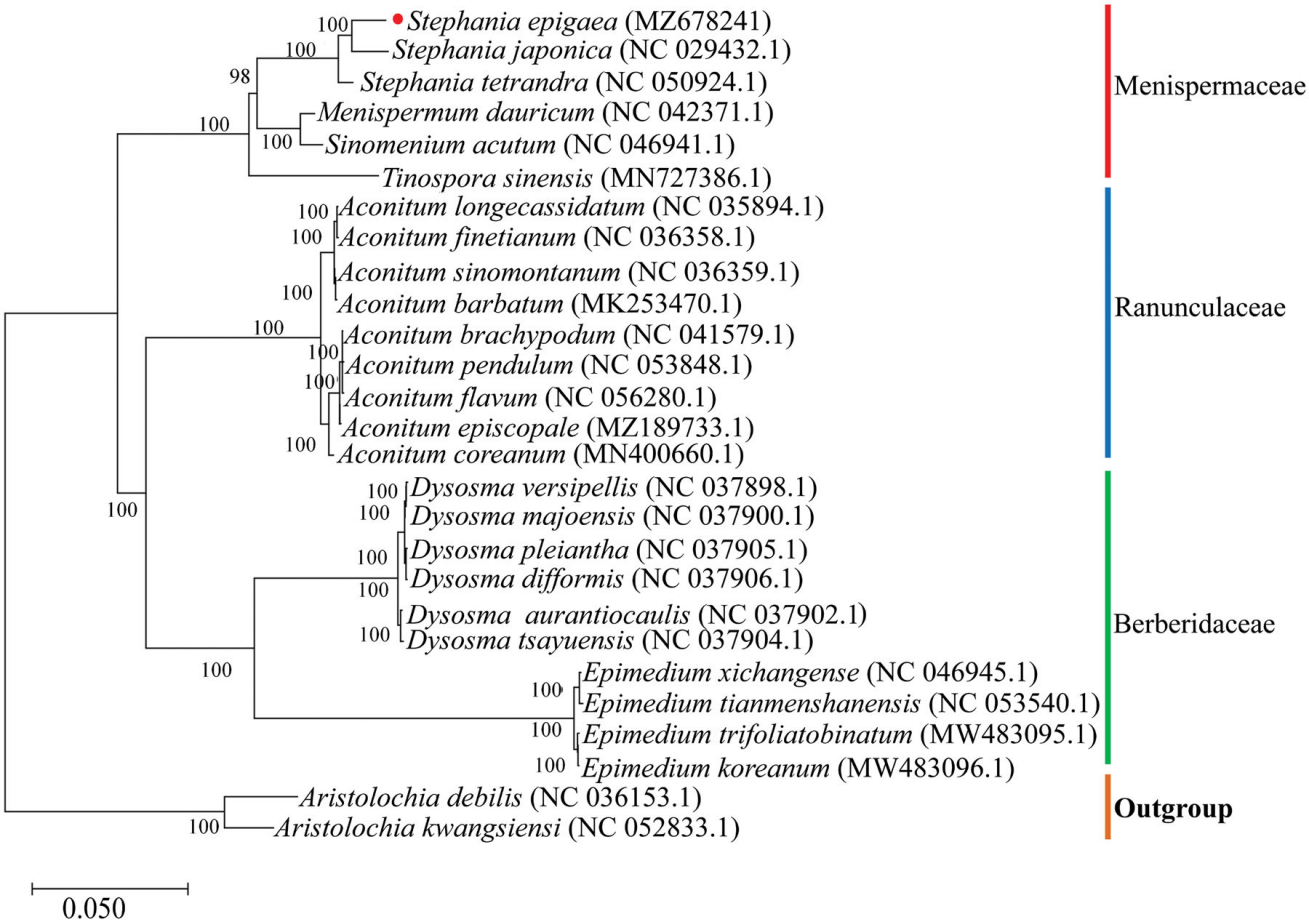
Phylogenetic tree reconstructed using the maximum-likelihood (ML) optimality criterion based on 26 chloroplast genome sequences with 1,000 bootstrap replicates.

## Data Availability

The genome sequence data, supporting the findings of this study, are openly available in GenBank of NCBI (https://www.ncbi.nlm.nih.gov) with the accession number is MZ678241, which permits unrestricted use, distribution and reproduction in any medium provided the original work is cited correctly. The associated BioProject, Bio-Sample and SRA numbers are PRJNA759697, SAMN21188258 and SRR15693952, respectively.
